# Production of viable male unreduced gametes in *Brassica *interspecific hybrids is genotype specific and stimulated by cold temperatures

**DOI:** 10.1186/1471-2229-11-103

**Published:** 2011-06-12

**Authors:** Annaliese S Mason, Matthew N Nelson, Guijun Yan, Wallace A Cowling

**Affiliations:** 1 School of Plant Biology M084 and The UWA Institute of Agriculture, The University of Western Australia, 35 Stirling Highway, Crawley, WA 6009, Australia

## Abstract

**Background:**

Unreduced gametes (gametes with the somatic chromosome number) may provide a pathway for evolutionary speciation via allopolyploid formation. We evaluated the effect of genotype and temperature on male unreduced gamete formation in *Brassica *allotetraploids and their interspecific hybrids. The frequency of unreduced gametes post-meiosis was estimated in sporads from the frequency of dyads or giant tetrads, and in pollen from the frequency of viable giant pollen compared with viable normal pollen. Giant tetrads were twice the volume of normal tetrads, and presumably resulted from pre-meiotic doubling of chromosome number. Giant pollen was defined as pollen with more than 1.5 *× *normal diameter, under the assumption that the doubling of DNA content in unreduced gametes would approximately double the pollen cell volume. The effect of genotype was assessed in five *B. napus*, two *B. carinata *and one *B. juncea *parents and in 13 interspecific hybrid combinations. The effect of temperature was assessed in a subset of genotypes in hot (day/night 30°C/20°C), warm (25°C/15°C), cool (18°C/13°C) and cold (10°C/5°C) treatments.

**Results:**

Based on estimates at the sporad stage, some interspecific hybrid genotypes produced unreduced gametes (range 0.06 to 3.29%) at more than an order of magnitude higher frequency than in the parents (range 0.00% to 0.11%). In nine hybrids that produced viable mature pollen, the frequency of viable giant pollen (range 0.2% to 33.5%) was much greater than in the parents (range 0.0% to 0.4%). Giant pollen, most likely formed from unreduced gametes, was more viable than normal pollen in hybrids. Two *B. napus *× *B. carinata *hybrids produced 9% and 23% unreduced gametes based on post-meiotic sporad observations in the cold temperature treatment, which was more than two orders of magnitude higher than in the parents.

**Conclusions:**

These results demonstrate that sources of unreduced gametes, required for the triploid bridge hypothesis of allopolyploid evolution, are readily available in some *Brassica *interspecific hybrid genotypes, especially at cold temperatures.

## Background

Unreduced gametes, or gametes with the somatic chromosome number (also referred to as "2*n*" gametes), are thought to play an important role in the evolution of polyploid species [1,2]. If two unreduced gametes unite, a fertile polyploid hybrid may form-either autopolyploid (fertilization within species) or allopolyploid (fertilization between species). Most plant species are now thought to be of recent or ancestral polyploid origin [3]. However, little is known about the frequency of unreduced gamete formation and the genetic and environmental factors which affect unreduced gamete production in most genera [2]. In *Solanum tuberosum *and *Trifolium pratense*, unreduced gamete production appears to be initiated by a monogenic recessive allele with other genes affecting the frequency of production (reviewed by Bretagnolle and Thompson (1995) [4]). Unreduced gamete-producing mutants linked to defects in the meiotic cell cycle machinery have also been recently identified in model plant *Arabidopsis thaliana*, leading to greater understanding of the mechanisms behind unreduced gamete formation [5]. However, little is known about the genetic or environmental factors that influence the production of unreduced gametes within most species, or in interspecific hybrid plants.

Interspecific hybrids tend to produce greater frequencies of unreduced gametes than their parents, as suggested by Ramsey and Schemske (1998) [2]. Unreduced gametes may be important in polyploid evolution via a triploid bridge [1]. A triploid bridge results from the union of an unreduced gamete (e.g. AA from species 2n = AA) with a reduced gamete (e.g. B from species 2n = BB). The triploid plant (AAB) may then produce unreduced gametes in a backcross with BB pollen to produce a new polyploid species (e.g. AAB + B = AABB). The triploid bridge hypothesis builds on the possibility that unbalanced interspecific hybrid plants produce more unreduced gametes than the parental species, but this has never been quantitatively tested under controlled experimental conditions [2]. The triploid bridge hypothesis may provide a more likely scenario for polyploid evolution than alternative hypotheses which require two unreduced gametes to unite by chance in an interspecific hybridization event (e.g. AA + BB = AABB), or which require chromosome doubling to occur in somatic tissue of a seed-derived hybrid (e.g. AB to AABB) [6].

Unreduced gamete production may be stimulated by stressful environmental conditions [2,7]. Cold spells in the field, cool glasshouse conditions and temperature cycling in growth chambers have all been implicated in increased unreduced gamete production (reviewed by Ramsey and Schemske (1998) [2] and briefly by Felber (1991) [8]). In *Rosa*, high temperatures induced spindle abnormalities causing an increase in unreduced pollen grain formation [9]. However, the interaction of temperature (or other environmental factors) and genotype on unreduced gamete production in interspecific hybrids has not been evaluated [2].

The *Brassica *"U's triangle" [10] species have valuable attributes for investigating the role of genotype and temperature on production of unreduced gametes in interspecific hybrids. U's Triangle includes three diploid species with genome complements AA, BB and CC (*B. rapa, B. nigra *and *B. oleracea *respectively) and three allotetraploid species AABB, AACC and BBCC (*B. juncea, B. napus *and *B. carinata *respectively). Interspecific trigenomic hybrids between the allotetraploid species (*B. juncea *× *B. napus*, AABC; *B. juncea *× *B. carinata*, BBAC; and *B. napus *× *B. carinata*, CCAB) may easily be created [11,12], and the hybrids often flower and produce viable gametes. The presence of one diploid genome (e.g. AA in AABC) in these unbalanced hybrids provides a moderate level of fertility [10,13], which is useful for assessing the production of unreduced gametes. Unreduced gametes have been observed in a number of *Brassica *interspecific hybrid types [14-18] including hybrids of the *Brassica *allotetraploids [19,20], although the frequency of unreduced gametes in parents and hybrids has never been quantified. No genetic or environmental factors influencing unreduced gamete production have been reported in *Brassica *species or their interspecific hybrids.

Most experiments on production of unreduced gametes have targeted male gametes [4], which are more easily assessed than female gametes. In dicotyledonous species, a structure known as a sporad is formed after meiosis in microspore mother cells, and this normally contains four daughter cells within an outer membrane and is known as a tetrad (Additional file 1). Sporads that contain unreduced gametes are of two types. The first type is a dyad, which contains two unreduced cells bound together within an outer cell membrane [21] (Additional file 1). The second is a giant tetrad, which contains four unreduced gametes [22]. Unreduced gametes are also expressed as "giant" pollen in some species (as reviewed by Bretagnolle and Thompson (1995) [4]) including *Brassica *[23], which is useful for assessment of the frequency of unreduced gametes and their viability.

In this study, we investigated genetic and temperature effects on male unreduced gamete production in interspecific hybrids between allotetraploid species in the *Brassica *triangle of U [10]. These species are ideal for this purpose since they produce hybrid plants that flower and many hybrids produce some viable male gametes. We evaluated male unreduced gamete production in five *B. napus*, two *B. carinata *and one *B. juncea *parental genotypes, and thirteen interspecific hybrid combinations among these parents. The effect of temperature during floral development on male unreduced gamete production was investigated in a subset of five parental genotypes and five interspecific hybrid combinations. Based on previous work [19,20], we hypothesized that the hybrids would have elevated frequencies of unreduced male gametes compared to their respective parents, and that this frequency would be influenced by genetic factors and by temperature.

## Results

### Characterization of putative interspecific hybrid plants

Seed set in the 34 possible *Brassica *interspecific cross combinations varied widely, and in 29 successful crosses there was an average of 0.82 seeds per pollinated bud (Table 1, Additional file 2). All three species were successful as male parents, but *B. carinata *was the least successful as a female parent (Table 1). The 90 putative hybrid plants from 23 combinations were assessed by genome-specific polymorphic simple sequence repeat markers, some of which were dosage-sensitive (see Nelson et al. (2009) [19] and Mason et al. (2011) [20] for details), and characterized for morphological attributes (Table 2). Of these, 79 plants were true hybrids resulting from a reduced (normal) gamete from both parents. Dosage-sensitive markers revealed four plants which were derived from an unreduced gamete from *B. napus *and a reduced gamete from *B. juncea *(Table 2, Additional file 3), and one plant which was derived from an aneuploid gamete from *B. carinata *and a reduced gamete from *B. juncea *(Table 2, Additional file 3). The remaining six plants were matromorphs (self-pollinated progeny from the maternal parent with the maternal parent phenotype) (Table 2). Another group of 40 putative hybrid plants were grown for the temperature experiment, and were all interspecific hybrids derived from a normal reduced gamete from both parents.

**Table 1:** Success of hand crossing between different genotypes of *B. napus, B. juncea *and *B. carinata*.

	Paternal							
Maternal	J1	C1	C2	N1	N2	N3	N4	N5
J1	-	0.18	0.22	2.47	2.51	4.49	1.77	1.74
C1	0.07	-	-	0.14	0.03	-	0.00	0.00
C2	0.00	-	-	0.03	0.07	-	0.03	0.02
N1	0.26	0.22	4.60	-	-	-	-	-
N2	0.13	0.36	1.00	-	-	-	-	-
N3	0.35	0.06	0.13	-	-	-	-	-
N4	0.74	0.13	0.57	-	-	-	-	-
N5	0.25	0.21	0.92	-	-	-	-	-

*B. napus *genotypes: N1, N2, N3, N4 and N5, *B. carinata *genotypes: C1 and C2 and *B. juncea *genotype: J1. Data are given as seeds per bud pollinated. Within-species combinations and *B. carinata *♀ × *B. napus *N3 ♂ crosses were not performed ("-").

**Table 2:** Genetic identity in an experimental interspecific hybrid plant population.

Species in cross	Genotype ♀ × ♂	No. plants total	True hybrids from molecular marker results, but with abnormal phenotype	Matromorphs (failed hybridity test, maternal phenotype)	True hybrids from molecular marker results and phenotype	Genotype ♀ × ♂	No. plants total	True hybrids from molecular marker results, but with abnormal phenotype	Matromorphs (failed hybridity test, maternal phenotype)	True hybrids from molecular marker results and phenotype
*B.*										
*juncea*	J1 × C1	15	1^a^	0	14	C1 × J1	1	0	1	0
&*B.*	J1 × C2	6	0	0	6					
*carinata*										

*B.*	J1 × N1	3	0	0	3	N1 × J1	9	0	0	9
*juncea*	J1 × N2	3	0	0	3	N2 × J1	3	1^b^	0	2
&*B.*	J1 × N3	3	0	0	3	N3 × J1	1	0	0	1
*napus*	J1 × N4	3	0	0	3	N4 × J1	1	0	1	0
	J1 × N5	7	0	0	7	N5 × J1	4	3^b^	0	1

	N1 × C1	5	0	0	5	C1 × N1	1	0	0	1
*B. *	N1 × C2	5	0	0	5	C2 × N1	1	0	0	1
*napus*	N2 × C2	3	0	0	3	C2 × N2	3	0	0	3
&*B.*	N3 × C1	1	0	0	1					
*carinata*	N4 × C1	6	0	4	2					
	N4 × C2	5	0	0	5	C2 × N4	1	0	0	1
	**Total**	**65**	**1**	**4**	**60**		**25**	**4**	**2**	**19**

^a ^missing some marker loci from *B. carinata *parent, presumed aneuploid gamete

^b ^Two copies of alleles from female parent to one copy of alleles from male parent, presumed unreduced female gamete.

Hybridity was confirmed using molecular marker analysis and phenotyping. True hybrids from molecular marker results which had abnormal phenotypes were further characterized using ten additional dosage sensitive molecular markers. *B. juncea *genotype "J1", *B. napus *genotypes "N1", "N2", "N3", "N4" and "N5" and *B. carinata *genotypes "C1" and "C2" were crossed to produce the experimental hybrid population, and a subset of the seeds produced sown out.

### Estimates of male unreduced gamete production through sporad observations

Sporads were classified according to the number of daughter cells present within the structure: monads, dyads, triads, tetrads, pentads, hexads and heptads. In addition, "giant sporads" were observed in some hybrids. These tetrads were disproportionately larger than other tetrads from the same anther. In order to estimate unreduced gamete formation from sporad observations, dyads were assumed to form two unreduced gametes, and giant sporads were assumed to produce four unreduced gametes [24]. Tetrads of normal size were assumed to produce four normal, reduced gametes. In order to estimate abnormal sporad production, monads, dyads, triads, pentads, hexads and heptads were assumed to form one, two, three, five, six and seven abnormal nuclei respectively.

All eight *B. juncea, B. napus *and *B. carinata *parent genotypes produced extremely low levels of unreduced gametes based on sporad observations (Table 3). Four dyads were observed out of more than 10 000 sporads in parent genotypes, equating to an overall unreduced gamete frequency of 0.04%. Dyads were only observed in 3/8 parent genotypes: *B. napus *N1 and N5 and *B. juncea *J1 (Table 3). In contrast, dyads were observed in all interspecific hybrid combinations (Table 4), and a few giant sporads were also observed in hybrid combinations *B. juncea *× *B. carinata *J1C1, *B. juncea *× *B. napus *J1N1 and *B. napus *× *B. carinata *N1C1 (Table 4). Average male unreduced gamete production in hybrids was estimated by sporad production at 1.32% (Table 4).

**Table 3:** Unreduced and abnormal male gamete production in amphidiploid *Brassica *species estimated by sporad counts.

Species	Genotype	No. plants	Total no. sporads observed	Total no. of abnormal sporads observed	Abnormal male gamete production	No. dyads observed	2n male gamete production*
*B. juncea*	J1	4	1916	1	0.03%	1	0.03%
*B. carinata*	C1	3	900	0	0.00%	0	0.00%
*B. carinata*	C2	5	2322	3	0.16%	0	0.00%
*B. napus*	N1	3	903	3	0.25%	2	0.11%
*B. napus*	N2	3	1230	0	0.00%	0	0.00%
*B. napus*	N3	2	700	0	0.00%	0	0.00%
*B. napus*	N4	2	600	0	0.00%	0	0.00%
*B. napus*	N5	5	1504	1	0.03%	1	0.03%

	**Total**	**27**	**10075**	**8**	**Av: 0.06%**	**4**	**Av: 0.02%**

* 2n male gamete production was estimated by the formula (number of nuclei in dyads)/(number of nuclei in all other sporad types)*100.

Both dyads and giant sporads were assumed to produce unreduced (2n) male gametes, whereas monads, dyads, triads, pentads, hexads and heptads were assumed to produce abnormal male gametes.

**Table 4:** Unreduced and abnormal male gamete production in interspecific hybrids of three amphidiploid *Brassica *species estimated by sporad counts.

Parental species in hybrid	Hybrid combination	No. plants	Total sporads	Abnormal sporads^†^	Abnormal male gametes (%)	Dyads	Giant sporads	2n male gametes (%)
*B. j × B. c*	J1C1	13	4579	79	1.97%	2	2	0.07%
*B. j × B. c*	J1C2	6	1812	10	0.74%	2	0	0.06%
*B. j × B. n*	J1N1	12	4346	292	6.17%	113	3	1.38%
*B. j × B. n*	J1N2	5	1710	202	9.33%	97	0	2.92%
*B. j × B. n*	J1N3	4	2255	209	5.94%	143	0	3.29%
*B. j × B. n*	J1N4	3	956	56	3.74%	36	0	1.93%
*B. j × B. n*	J1N5	7	2197	97	2.72%	73	0	1.69%
*B. n × B. c*	N1C1	6	2417	85	2.45%	52	10	1.51%
*B. n × B. c*	N1C2	6	1911	111	5.46%	40	0	1.05%
*B. n × B. c*	N2C2	6	2108	68	2.23%	46	0	1.10%
*B. n × B. c*	N3C1	1	301	1	0.17%	1	0	0.17%
*B. n × B. c*	N4C1	2	609	9	0.95%	6	0	0.50%
*B. n × B. c*	N4C2	6	2261	155	5.43%	68	0	1.53%
	**Total**	**77**	**27462**	**1374**	Av: 3.64%***	**679**	**15**	Av: 1.32%**

** Significant differences between genotypes (p < 0.01, one-way ANOVA)

***Significant differences between genotypes (p < 0.001, one-way ANOVA)

^† ^Both dyads and giant sporads were assumed to produce unreduced (2n) male gametes, and monads, dyads, triads, pentads, hexads and heptads and giant sporads were assumed to produce abnormal male gametes.

Hybrids were produced between five doubled-haploid derived genotypes of *B. napus *(*B. n*: N1, N2, N3, N4 and N5), two doubled-haploid derived genotypes of *B. carinata *(*B. c*: C1 and C2) and one near-homozygous inbred genotype of *B. juncea *(*B. j*: J1). Interspecific hybrid combinations are given as a combination of parent codes. Hybrid combinations with different maternal parent but the same parent genotypes were pooled after the model unreduced gametes ~ genotype + maternal parent revealed no significant effect of maternal parent on unreduced gamete production.

Hybrid combinations varied in the frequency of total abnormal sporads, and the derived estimate of unreduced gamete production at the sporad stage ranged from 0.06% in *B. juncea *× *B. carinata *J1C2 to 3.3% in *B. juncea *× *B. napus *J1N3 (Table 4). There was no significant effect of maternal parent (cytoplasm) on unreduced gamete production as estimated by sporad observations, based on linear mixed models. Overall, interspecific hybrid combinations produced more unreduced gametes (average 1.32%) as estimated from sporad observations than their parent cultivars (average 0.02%) (Table 3, Table 4).

### The effect of temperature on unreduced gametes observed at the sporad stage

**Figure 1:**
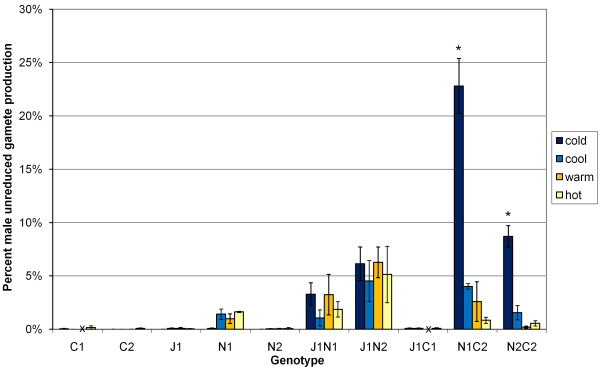
**Male unreduced gamete production in two *B. carinata *lines (C1 and C2), one *B. juncea *line (J1), two *B. napus *cultivars (N1 and N2) and in the interspecific hybrids between them at four different temperatures**. Unreduced gamete production was assessed by counts of dyads and giant sporads at the sporad stage of pollen development. Temperature treatments were (day 12 h/night 12 h) as follows: hot: 30°C/20°C, warm: 25°C/15°C, cool: 18°C/13°C, cold: 10°C/5°C. Data are given as group averages with ± one standard error bars. J1C1 and C1 plants under the "warm" growth condition died before flowering, and these missing values are indicated by an "x" on the x-axis. * Indicates significant difference (p < 0.001) between that temperature treatment and other temperature treatments for that genotype.

**Figure 2:**
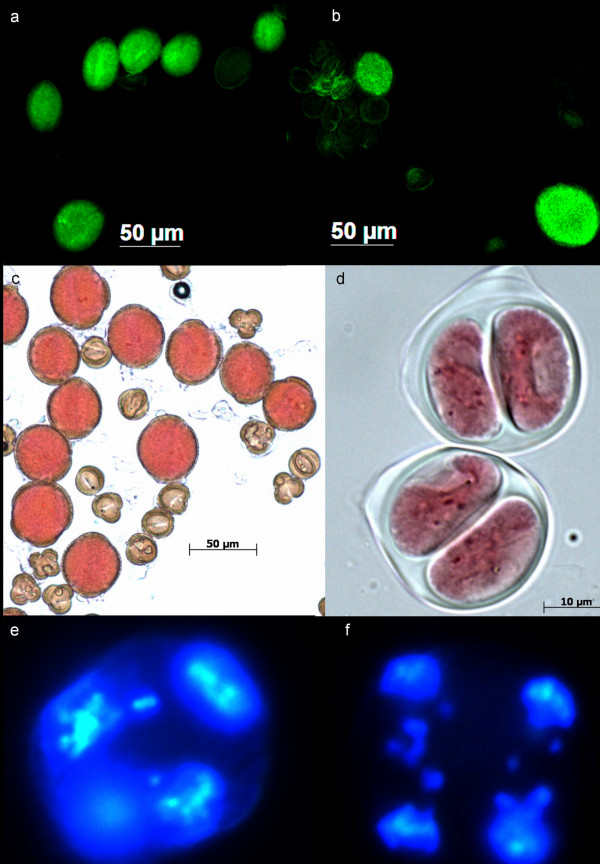
**Male unreduced gamete formation in *Brassica***. a) A "giant" pollen grain in *B. napus *and several normal sized pollen grains; b) Putative viable unreduced (large, bright), viable reduced (small, bright) pollen and non-viable (shrunken, dull) pollen in an interspecific hybrid; c) *B. napus *× *B. carinata *(CCAB) pollen in cold (10°C day/5°C night) temperature); d) Two dyads produced by a *B. napus *× *B. carinata *(CCAB) hybrid in cold (10°C day/5°C night) temperature; e) beginning of telophase II in an interspecific hybrid, showing a tetrahedral nuclei arrangement within the cell as a result of normal, perpendicular spindle orientation, but with laggard chromosomes outside the nuclei and f) Anaphase II showing parallel spindles, a common mechanism of dyad formation in *Brassica*.

Parental genotypes J1, N2, C1 and C2 and *B. juncea *× *B. carinata *J1C1 averaged less than 0.2% unreduced male gametes across all temperature treatments, as estimated from sporad observations (Figure 1). The average unreduced gamete production across temperature treatments of *B. juncea *× *B. napus *J1N1 and J1N2 (2.4% and 5.5%, respectively) was much larger than in the parent genotypes (J1: 0.05%, N1: 1.03% and N2: 0.04%) but there was no apparent effect of temperature on these hybrids (Figure 1). However, *B. napus *× *B. carinata *N1C2 and N2C2 produced 23% and 9% unreduced gametes respectively in the cold temperature treatment (Figure 1, Figure 2c, d), which was more than two orders of magnitude greater than in the parent species. Giant viable pollen was visibly prevalent in these hybrid genotypes under cold temperatures (Figure 2c).

### Viable pollen in hybrids and parents

**Figure 3:**
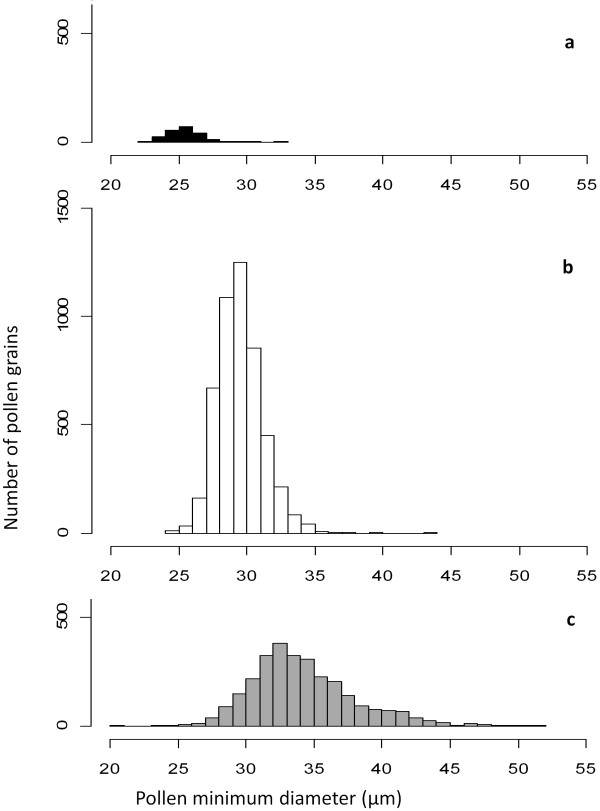
**Viable pollen size distributions and ploidy in parental lines and cultivars of Brassica**. Pollen viability was estimated using fluorescein diacetate stain and pollen diameter was measured under the microscope in μm (viable pollen only), with the expectation that pollen size would be proportional to DNA content of the pollen grain. a) *B. rapa *(2*n *= 2*x *= AA) pollen, expected pollen ploidy *n *= *x = *A; b) *B. juncea *(2*n *= 4*x *= AABB), *B. napus *(2*n *= 4*x *= AACC) and *B. carinata *(2*n *= 4*x *= BBCC) pollen, expected pollen ploidy *n *= 2*x *= AB, AC or BC respectively; c) 2*n *= 4x interspecific hybrid *B. juncea × B. napus *(AABC), *B. juncea × B. carinata *(BBAC) and *B. napus × B. carinata *(CCAB) pollen, expected ploidy for reduced pollen n = *x *- 3*x*: A-ABC, B-ABC and C-ABC respectively. The bias of the hybrid pollen size distribution to the right suggests unreduced gamete production (ploidy 4*x *and above) as well as a viability advantage of higher DNA contents (mean of distribution > 2*x*, expected ploidy distribution *x *- 3*x*).

Viable pollen in hybrids was on average larger (34.2 μm minimum diameter) than viable pollen in parent species (29.5 μm), with a greater size range (20.6 μm to 51.9 μm) (Figure 3) and more spherical shape. There were small but significant differences in average pollen diameter between genotypes. *B. napus *and *B. carinata *genotypes averaged 28.5 to 29.5 μm, and the *B. juncea *genotype averaged 31.7 μm diameter.

Giant pollen grains were observed very infrequently in the parents (Table 5, Figure 2a). A maximum of two giant viable pollen grains were observed per parent genotype across 29 plants (Table 5). "Giant" pollen grains were defined as viable pollen grains with a minimum diameter greater than 1.5 times the genotype mean in the parent genotypes, and in interspecific hybrid combinations as 1.5 times the reduced (2*x*) pollen mid-parent mean diameter of the two parent genotypes of that hybrid. This represents approximately double the volume of reduced gametes. Viable giant pollen was observed in all nine interspecific hybrid combinations which produced viable pollen (*B. juncea *× *B. carinata *J1C1, Table 6, Figure 2b). The frequency of giant pollen production varied significantly between interspecific hybrid genotypes (Table 6). *Brassica juncea *× *B. carinata *hybrids produced significantly less giant pollen (as measured in the viable pollen fraction) than other hybrid types (0.2% to 1.8%, Table 6). *B. juncea *× *B. napus *J1N2 and *B. napus *× *B. carinata *N1C2 produced the most giant pollen as a fraction of viable pollen (30% to 34%, Table 6), while the remaining genotypes fell in between the two extremes (6% to 19%, Table 6). Overall, interspecific hybrids produced significantly more giant pollen than their parents (p < 0.01, Student's t-test; Table 5, Table 6).

**Table 5:** "Giant" pollen observation in amphidiploid *Brassica *species.

Genotype	Species	No. of plants	Total viable pollen measured	Giant pollen observed	Giant pollen as a percentage of viable pollen
J1	*B. juncea*	5	653	1	0.15%
N1	*B. napus*	2	386	0	0.00%
N2	*B. napus*	5	1001	0	0.00%
N3	*B. napus*	3	515	2	0.39%
N4	*B. napus*	3	885	1	0.11%
N5	*B. napus*	2	419	0	0.00%
C1	*B. carinata*	4	279	1	0.36%
C2	*B. carinata*	5	528	1	0.19%
	**Total**	**29**	**4666**	**6**	**Av: 0.15%**

A pollen grain was determined to be "giant" if the minimum diameter of the pollen grain exceeded 1.5 × the mean pollen diameter observed in pollen production by that plant. No significant differences in giant pollen production were observed between genotypes.

**Table 6:** "Giant" pollen observations in *Brassica juncea *× *B. napus *(AABC), *B. juncea *× *B. carinata *(BBAC) and *B. napus *× *B. carinata *(CCAB) hybrids.

Parental species in hybrid	Hybrid combination	No. of plants	Average pollen viability^Ɨ^	Average self-pollinated seed set	Total viable pollen measured	Giant pollen	Giant pollen (% of viable pollen)^Ɨ^
*B. j × B. c*	J1C1	14	6%^ab^	99	443	1	0.2%^a^
*B. j × B. c*	J1C2	6	7%^ab^	127	626	11	1.8%^a^
*B. j × B. n*	J1N1	8	14%^b^	0	353	21	5.9%^b^
*B. j × B. n*	J1N2	4	4%^ab^	6	227	76	33.5%^c^
*B. j × B. n*	J1N3	4	12%^ab^	3	524	50	9.5%^b^
*B. j × B. n*	J1N4	3	9%^ab^	2	372	55	14.8%^b^
*B. j × B. n*	J1N5	8	26%^c^	4	208	20	9.6%^b^
*B. n × B. c*	N1C1	1	1%^a^	0	21	4	19.0%^abc^
*B. n × B. c*	N1C2	4	2%^a^	3	86	26	30.2%^c^
*B. n × B. c*	N2C2	6	0%^a^	0	0	-	-
*B. n × B. c*	N3C1	1	0%^abc^	0	0	-	-
*B. n × B. c*	N4C1	2	0%^ab^	0	0	-	-
*B. n × B. c*	N4C2	6	0%^a^	0	0	-	-
	**Total**	**67**	Av: 6%***	19***	**2860**	**264**	Av: 13.8%***

*** Significant differences between genotypes (p < 0.001, one-way ANOVA)

^Ɨ ^Numbers in the same column followed by the same letters are not significantly different (pairwise t-tests with Holm p-adjustment method for multiple comparisons).

Hybrids were produced between five genotypes of *B. napus *(*B. n*: N1, N2, N3, N4 and N5), two genotypes of *B. carinata *(*B. c*: C1 and C2) and one genotype of *B. juncea *(*B. j*: J1). Hypothetical "giant" pollen size in the hybrids was estimated under the assumptions that a) doubling DNA content would double pollen grain volume, and b) that reduced pollen in hybrids would have a maximum DNA content of 1.5 times parent (2*x*) DNA content. Hybrid combinations with different maternal parent but the same two parent genotypes were pooled after the model unreduced gametes ~ hybrid genotype + maternal parent revealed no significant effect of maternal parent on unreduced gamete production.

### Estimation of unreduced gametes derived from sporads and viable pollen

The frequency of unreduced gametes in hybrids, as estimated from the proportion of viable giant pollen compared with total viable pollen (average 13.8%, Table 6) was much higher than estimates based on observations of sporads (average 1.32%, Table 4) in interspecific hybrids (p < 0.05). However, there was a high proportion of pollen grains in hybrids that were not viable. Consequently, giant pollen as a fraction of total pollen production (including shrunken, non-viable pollen grains) was 1.22%, which was similar to estimates of unreduced nuclei at the sporad stage. There was no difference between these two measures of male unreduced gamete frequency in the *B. juncea, B. napus *and *B. carinata *parent genotypes.

### Evidence of meiotic abnormalities

Abnormal sporads (other than dyads and giant sporads) were also observed, including monads, triads, pentads, hexads and heptads. These were assumed to contain gametes with abnormal chromosome numbers. Abnormal sporad production in all 27 *B. juncea, B. napus *and *B. carinata *plants at 18°C/13°C day/night temperature was extremely low, ranging from 0% to 0.25% (Table 2). Hybrid plants produced abnormal sporads with a frequency ranging from 0.2% to 6.2% (Table 4). Triads, pentads and hexads had nuclei with variable size: almost all pentads and hexads showed four large nuclei and one and two extra small nuclei respectively. Pentad and hexad frequencies were highly positively correlated (r^2 ^= 0.56, p < 0.0001), and triad and dyad frequencies were also positively correlated across hybrid plants (r^2 ^= 0.26, p < 0.0001), but there was no significant relationship among other sporad types. Some chromosomes were observed to be excluded from nucleus formation at telophase II, and multiple chromosomes were often observed as laggards at anaphase II (Figure 2e). Parallel spindles (a meiotic phenomenon leading to unreduced gamete formation) were also observed in some hybrid genotypes (Figure 2f).

Hybrid genotype *B. napus *× *B. carinata *N1C2 produced significantly more sporads with more than four nuclei (pentads, hexads and heptads) in the hot temperature treatment (11%) than in the warm (3%), cool (1%) and cold (0.5%) temperature treatments. *Brassica napus *N1 also produced more sporads with more than four nuclei (9%) in the hot temperature treatment compared to the other temperature treatments (1%). The synchronous timing of meiosis was also deregulated in *B. carinata *C1, *B. napus *N1 and *B. juncea *× *B. napus *J1N1 in response to the hot temperature treatment, with many stages of meiosis from prophase I to sporads often present in the same anther (results not shown). *Brassica juncea *× *B. napus *J1N1 also exhibited asynchronous meiotic divisions in the warm temperature treatment.

### The effects of genotype and temperature on pollen viability

Hybrid combinations varied significantly in pollen viability and seed set (Table 6). All *B. juncea *× *B. napus *(AABC) and *B. juncea *× *B. carinata *(BBAC) genotypes produced some viable pollen (4% to 25% on average by genotype, Table 6). However, all six *B. napus *× *B. carinata *(CCAB) hybrid genotypes had < 2% viable pollen, and four of these were male-sterile (Table 6). *Brassica juncea *× *B. napus *(AABC) hybrids produced the most viable pollen (Table 6), but *B. juncea *× *B. carinata *(BBAC) hybrids produced the most self-pollinated seed (13 to 248 per plant, Table 6).

**Figure 4:**
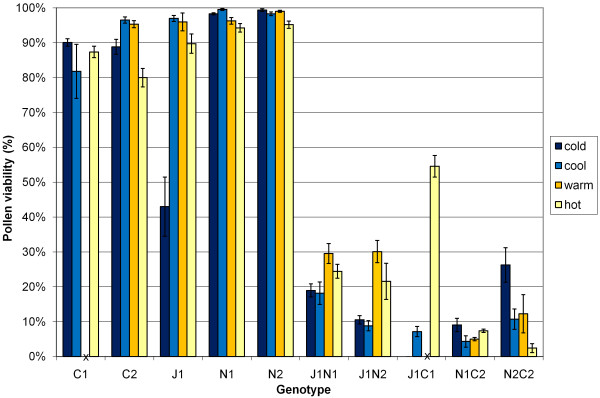
**Pollen viability estimates for five *Brassica *parent lines and cultivars (J1 -* B. juncea*, N1 and N2 - *B. napus*, C1 and C2 -* B. carinata*) and five *Brassica *interspecific hybrid genotypes at four different temperature treatments at 12 h day/night temperatures-hot (30°C/20°C), warm (25°C/15°C), cool (18°C/13°C) and cold (10°C/5°C)**. Interspecific hybrid genotypes J1N1 and J1N2 are *B. juncea *× *B. napus *hybrids from two different *B. napus *parent cultivars, J1C1 a *B. juncea *× *B. carinata *hybrid and N1C2 and N2C2 *B. napus *× *B. carinata *hybrids from the same two *B. napus *cultivars. J1C1 and C1 plants under the "warm" growth condition died before flowering, and these missing values are indicated by an "x". Data are given as group averages with ± one standard error bars.

Most interspecific hybrids produced at least some flowers with developed anthers and viable pollen in all (10°C/5°C, 18°C/10°C, 25°C/15°C and 30°C/20°C) temperature treatments. However, *B. juncea *× *B. carinata *J1C1 hybrids produced entirely male-sterile flowers in the cold temperature treatment (10°C/5°C day/night) (Figure 4), and the majority of flowers produced by both *B. napus *× *B. carinata *genotypes in the hot temperature treatment were also male-sterile (Figure 4). Some male-sterile flowers were also produced by *B. napus *× *B. carinata *genotypes under the warm and cool temperature treatments, and by *B. carinata *C2, *B. napus *N1 and *B. juncea *× *B. napus *hybrids J1N1 and J1N2 under the hot temperature treatment. Pollen viability in the parent genotypes was not significantly affected by temperature treatment, with two exceptions: *B. juncea *J1 pollen viability was lower in the cold treatment (Figure 4), and *B. carinata *C2 pollen viability was lower in the hot treatment (Figure 4). *Brassica juncea *× *B. carinata *J1C1, *B. juncea *× *B. napus *J1N2 and *B. napus *× *B. carinata *N2C2 pollen viability was also affected by temperature (Figure 4, Figure 2c).

Flowering time in most interspecific hybrids was intermediate between their maternal and paternal parent varieties across all temperature treatments in the temperature experiment (Additional file 4). The cold temperature treatment delayed flowering by 40 days on average within the temperature experiment (Additional file 4).

## Discussion

The frequency of unreduced gametes produced by some *Brassica *interspecific hybrids exceeded the frequency in parental genotypes by more than one order of magnitude (Table 3, Table 4), and there was significant variation among genotypes (Table 4). At cold temperatures, some genotypes produced unreduced male gametes at two orders of magnitude higher level than in the parents (Figure 1). The frequency of viable giant pollen from unreduced gametes, as a proportion of total viable pollen, was high in hybrids due to the low viability of reduced pollen in hybrids. Under these conditions, viable unreduced gametes would be readily available for polyploid species evolution *via Brassica *interspecific hybrids, as required by the triploid bridge hypothesis of allopolyploid evolution [1,2].

High temperature did not stimulate formation of unreduced gametes in any parental or hybrid genotypes. The parental genotypes produced very low frequencies of unreduced gametes (Table 3, Table 5), as expected from established species (even allopolyploid species) with diploidized meiosis [3]. The interspecific hybrid genotypes had unbalanced genome complements (one diploid and two haploid genomes) most likely with univalent chromosomes at meiosis [25], which may be associated with the increased formation of unreduced male gametes in these hybrid types. The relatively low level of unreduced gametes observed in *B. juncea *× *B. carinata *(BBAC) hybrids (known to have fewer univalents than *B. napus *× *B. juncea *(AABC) and *B. napus *× *B. carinata *(CCAB) types; [25,26]) supports this hypothesis. However, different genotypes of *B. napus *× *B. juncea *(AABC) and *B. napus *× *B. carinata *(CCAB) hybrids produced a wide range of frequencies of unreduced gametes under the same conditions (Figure 1, Table 6), which indicates that genetic factors inherited from parent species mediate the production of unreduced gametes.

The triploid bridge hypothesis of allopolyploid evolution has recently gained support [3,6,27,28]. The triploid bridge hypothesis suggests that unreduced gamete YY from a diploid species with genome complement YY unites with reduced gamete Z from a diploid species with genome complement ZZ to give triploid hybrid YY+Z = YYZ [2]. This triploid hybrid then produces unreduced gamete YYZ which unites with reduced gamete Z from parent species ZZ to give new balanced polyploid YYZ + Z = YYZZ. A key factor in the triploid bridge hypothesis of allopolyploid evolution is the production of unreduced gametes by the interspecific hybrid [2]. Our results show that unreduced gamete production by *Brassica *interspecific hybrids is higher than in their parent genotypes, which will promote polyploid evolution via a triploid bridge.

The hybrid pollen size distribution, expected to be distributed around a predicted 2*x *mean pollen size, was biased to the right (> 2*x*) in our experiment (Figure 3). This suggests that loss of univalent chromosomes conferred a viability penalty for gametes produced by the interspecific hybrids. Unreduced gametes were also more viable during pollen development than reduced gametes produced by the interspecific hybrids in our experiment, as the fraction of unreduced gametes estimated in the viable pollen fraction was much greater (13.8%) than the fraction of unreduced gametes estimated in the sporad population (1.32%). This supports a similar finding of high viability of male unreduced gametes in *Arabidopsis *[27]. We also observed selection of unreduced gametes in the initial crossing event to produce four "triploid" hybrids with a diploid genome from *B. napus *and a haploid genome from *B. juncea *(Table 1). This suggests that unreduced gametes may be more viable in all interspecific crosses irrespective of ploidy level. Mechanisms of polyploidization and speciation (such as unreduced gamete production) are expected to be conserved with increasing ploidy [29], as evidenced by the multiple rounds of polyploidy found in most species [30]. Hence, unreduced gamete production by interspecific hybrids among *Brassica *allotetraploids may be expected to mimic processes of unreduced gamete production in diploid *Brassica *interspecific hybrids. Interestingly, Palmer et al. (1983) [31] predicted from chloroplast DNA analysis that back-crossing of a novel hybrid to the paternal parent population must have occurred several times during the evolution of *B. napus *from progenitor species *B. rapa *and *B. oleracea*, supporting the triploid bridge mechanism of polyploid formation in this genus.

Abnormal sporad production is predicted to be the result of three mechanistic processes from our study: laggard chromosomes, abnormal spindle formation and pre-meiotic doubling. Firstly, pentad and hexad production were highly positively correlated (r^2 ^= 0.56), and most sporads of this form appeared to have four larger nuclei and one or two small nuclei. These extra nuclei are probably formed by laggard chromosomes at meiosis (Figure 2e, also suggested by d'Erfurth et al. (2008) [27]), which form micronuclei visible at the sporad stage (also occasionally detected as very small, non-staining cells at the pollen stage, data not shown). The correlation between dyad and triad frequency observed in our experiment may be due to a shared meiotic mechanism. The most likely meiotic mechanism that accounts for both dyads and triads is abnormal spindle formation. Several major gene mutations in *Brassica *relative *Arabidopsis *result in high frequencies of dyads and triads through the same mechanism of parallel spindles at meiosis II (Additional file 1) [5,27,32]. A single gene is thought to be responsible in *Solanum *for fused, parallel and tripolar spindles [33], which may give rise to dyads, dyads and triads respectively. If a single gene is also responsible for abnormal spindle orientation in *Brassica*, this may explain the correlation between dyads and triads observed in our experiment. Finally, the occasional observation of "giant" sporads in our study (also observed in *Brassica *by Fukushima (1930) [24]) suggests that somatic doubling of some pollen mother cells may occur prior to meiosis, although possible causes of this effect are not known.

Temperature had two different effects on meiotic behavior as assessed by meiotic products at the sporad stage in our study. Firstly, the cold temperature treatment stimulated unreduced gamete production in *B. napus *× *B. carinata *interspecific hybrid combinations N1C2 and N2C2 (Figure 3). Secondly, the hot temperature treatment appeared to stimulate abnormal meiosis in *B. napus *genotype N1 and in *B. napus *× *B. carinata *N1C2. Meiosis was poorly synchronized within each anther and frequently resulted in additional nuclei or micronuclei, probably as a result of chromosome laggards or spindle abnormalities. Chromosome synapsis in meiosis has long been known to be influenced by temperature [34,35]. Recent studies in *Arabidopsis *and yeast have implicated chromatin remodeling in response to cool temperatures, resulting in physical blocks to gene transcription [36,37]. DNA methylation has also been implicated in the cool temperature vernalization response for a number of plant species [38]. As the heat and cold treatments used in this study (30°C day/20°C night and 10°C day/5°C night) could potentially be reached in normal growing conditions worldwide for *Brassica*, this highlights the need for further investigation of the role of meiotic response to temperature in polyploid fertility, speciation and establishment.

## Conclusions

Unreduced gametes were produced at an order of magnitude higher on average in some interspecific hybrids compared to their parent genotypes. Unreduced gametes were also more viable than reduced gametes in interspecific hybrids. Genotypic variation was present among hybrid combinations in the production of unreduced gametes in *Brassica *interspecific hybrids, and some hybrid genotypes were stimulated by cold temperatures to produce high levels of unreduced gametes. These results demonstrate that a source of unreduced gametes, required for the triploid bridge hypothesis of allopolyploid species formation, is readily available in *Brassica *interspecific hybrids especially if cold temperatures are present during flowering.

## Methods

### Plant material

In this study, parent genotypes were derived from a process of doubled-haploidy through microspore culture protocols described in Nelson et al. (2009) [19] and Cousin and Nelson (2009) [39] and bulked by pure seed methods. The five *B. napus *genotypes were "Surpass400_024DH", "Trilogy", "Westar_010DH", "Monty_028DH" and "Boomer", and are hereafter referred to as N1, N2, N3, N4 and N5, respectively. The two *B. carinata *genotypes were "195923.3.2_01DH" and "94024.2_02DH", and are hereafter referred to as C1 and C2, respectively. Inbred *B. juncea *parent line "JN9-04" (hereafter referred to as J1) was a selfed single plant selection by Janet Wroth (UWA, Perth, Australia) from near canola-quality *Brassica juncea *line "JN9" supplied by Wayne Burton (Department of Primary Industries, Horsham, Victoria, Australia).

Interspecific hybrid combinations were made between parental genotypes of *B. juncea, B. napus *and *B. carinata *by hand emasculation and pollination in a controlled environment room (CER) at 18°C/13°C day/night with a 16 h photoperiod at a light intensity of approximately 500 μmol m^-2^s^-1^. Each cultivar or line of one species was crossed with every cultivar or line of the other two species (Table 1), and all reciprocal crosses were also attempted. At least 16 (average 59) buds were pollinated for each cross combination in each direction (Additional file 2). Interspecific hybrid combinations are hereafter referred to by the two parent genotype codes (e.g. J1N1 = *B. juncea *J1 *× B. napus *N1 hybrid, with J1 as female parent). Cross-pollination was prevented by enclosing racemes in bread bags.

### Growth conditions and experimental design

A subset of the putative hybrid seed was planted out in two groups to generate the experimental interspecific hybrid populations. In the first group, ninety putative hybrid seeds were germinated and grown to maturity, representing 23 hybrid combinations, of which 21 combinations gave true hybrid plants as confirmed by molecular marker analysis (for molecular marker methods, see below) (Table 1). There were 13 successful combinations of parental genotypes, including 10 for which the reciprocal was also successful (see Table 1): five *B. juncea × B. napus *(J1N1, J1N2, J1N3, J1N4 and J1N5), two *B. juncea × B. carinata *(J1C1 and J1C2) and six *B. napus × B. carinata *(N1C1, N1C2, N2C2, N3C1, N4C1 and N4C2). Selfed seed of each amphidiploid parent genotype was germinated and grown to maturity at the same time as the hybrid seeds. Seeds from most hybrid combinations and parent genotypes were germinated in potting mix and grown in pots in a controlled environment room (CER) at 18°C/13°C day/night with a 16 h photoperiod at a light intensity of approximately 500 μmol m^-2^s^-1^. For cross combinations and reciprocals which yielded only a single seed (*B. carinata × B. napus *C1J1, C1N1, C2N1 and C2N4; *B. napus × B. carinata *N3C1), the seeds were germinated on agar under sterile conditions in Petri dishes before being transferred to soil in the CER for growth and subsequent measurements. Twelve of the fifteen plants in progeny set *B. juncea × B. carinata *J1C1 were transferred at the two-to four-leaf stage to a glasshouse with evaporative cooling in the spring of 2008 at The University of Western Australia (Perth, Australia). Morphology, sporad production, pollen viability, pollen size measurements for viable pollen grains and self-seed set data were collected for all hybrid combinations and parent controls. Reciprocals were pooled due to low numbers after the linear mixed model: unreduced gamete production ~ genotype + maternal parent showed no significant effect of maternal parent (p > 0.05). Pollen viability was estimated using fluorescein diacetate stain using methods detailed in Heslop-Harrison et al. (1984) [40]. Only pollen grains which fluoresced brightly (indicating an intact cell membrane) were assumed to be viable and subsequently measured. Pictures were taken of pollen using an AxioCamMR3 microscope camera (Carl Zeiss, Germany) and measurements made of pollen minimum diameter using Axiovision software v4.6.3 (Carl Zeiss Imaging Solutions GmbH, 2007). Self-pollination was promoted by enclosing plants in bread bags at flowering before collecting seeds.

A subset of hybrid combinations and parent genotypes with a wide range of unreduced gamete production was selected to test the effect of temperature on male unreduced gamete production: five interspecific hybrid combinations (*B. juncea × B. napus *J1N1, J1N2; *B. juncea × B. carinata *J1C1; *B. napus × B. carinata *N1C2, N2C2) and their five parent genotypes (*B. juncea *J1; *B. napus *N1, N2; *B. carinata *C1, C2). These plants were all grown under a 12 h photoperiod at a light intensity of approximately 650 μmol m^-2^s^-1^. Seeds were planted in large shared pots for each genotype in a CER at 18°C/10°C day/night, and two seedlings of each genotype were transferred to individual 15 cm deep pots in four trays (4 × 5 cell) at the two to six leaf stage in a randomized design. After five weeks (just before bolting in the earliest genotypes) one tray was moved to 30°C/20°C day/night (hot), one tray to 25°C/15°C day/night (warm) and another tray to 10°C/5°C day/night (cold), with one tray remaining at 18°C/10°C (cool). Five plants in the "warm" temperature treatment died before flowering and were recorded as missing values: 2 × *B. carinata *C1, 2 × *B. juncea × B. carinata *J1C1 and 1 × *B. juncea *J1. Pollen viability estimates and sporad counts were performed using 1% acetic acid carmine stain. Mature, swollen pollen grains strongly staining red were assumed to be viable. At least 300 pollen grains from each of two different flowers were counted for each plant. In plants which produced both male sterile and male fertile flowers, only male fertile flowers were assessed for pollen viability. Flowering time was recorded as days to first floral bud opening.

### Sporad observations

Male unreduced gamete production was estimated by assessment of meiotic products at the sporad stage (Additional file 1). Sporads were classified as monads, dyads, triads, tetrads, pentads, hexads or heptads according to the number of nuclei present (1 to 7 nuclei, respectively). "Giant" sporads were identified visually as being disproportionately larger tetrads compared to all surrounding tetrads from the same anther, in particular containing much larger nuclei.

Male unreduced gamete frequencies were estimated using the formula:

Monads, dyads, triads, pentads and hexads were assumed to produce abnormal (aneuploid, diploid or tetraploid) gametes.

Abnormal gamete frequency was calculated from the formula:

At least 300 sporads per bud were assessed for each plant, and two different buds were examined for each plant in the temperature study.

### Pollen size estimates

Pollen size was also used to estimate viable unreduced gamete production in the first experimental population, under the assumption that doubling DNA content would result in doubling pollen volume. Pollen length and width were measured on a subset of pollen grains (estimated to be viable using fluorescein diacetate stain) from each parent genotype, and these dimensions used to calculate pollen grain volume (based on volume calculations using diameter and length for an ovoid). Pollen from diploid species *B. rapa *"Chiifu" (2*n *= AA = 2*x*) was also measured as a control. Mid-parent mean was used as the value to calculate expected pollen size in the interspecific hybrids, to control for genotypic effects. Heyn (1977) [22] demonstrated that "giant" pollen diameter in *B. nigra *(2*n *= BB = 2*x*) ranged from 1.26 to 1.54 times the diameter of normal, reduced pollen (genome complement B = 1*x*). Following Heyn (1977) [22], we used a minimum value of 1.5 times "normal" reduced (2*x*) pollen diameter to classify the pollen as "giant" (4*x *or greater ploidy, e.g. AABC). The average minimum diameter for classifying pollen as 4*x *in this study was 39.7 μm.

### DNA extractions and molecular marker analysis

DNA was extracted from leaf tissue using a MagAttract 96 DNA Plant Core Kit (Qiagen). Hybridity testing was carried out using microsatellite molecular markers sJ0338 and sJ6846 (B-genome microsatellite markers with known genomic locations in *B. juncea *provided by A. Sharpe and D. Lydiate [Agriculture and AgriFood Canada Saskatoon Research Centre, Saskatoon; pers. comm.; for more information, see http://brassica.agr.gc.ca]). Both markers produced a band unique to two of the three parent species. Plants which were confirmed as hybrids from molecular marker results but which did not show an intermediate phenotype between parent genotypes were further characterized using ten dosage sensitive markers amplifying 34 species-specific microsatellite marker alleles in the A, B and C genomes (sNRD03, sN11707, sN11722, sS2066, sN1988, sS1949, sR12384I, sR10417, sR12387, sN13039), to determine the relative number of parent genomes present. Fragment amplification using fluorescently-labeled primers and visualization using an AB3730*xl *capillary sequencer (Applied Biosystems) was carried out according to the methods detailed in Nelson et al. (2009) [20].

### Statistical tests

Statistical analyses were carried out using R version 2.10.1 (The R Foundation for Statistical Computing, 2009). Figures were generated using Microsoft Excel 2002 (Microsoft Corporation). Pairwise t-tests with pooled SD and Holm p-value adjustment method for multiple comparisons were used for post-hoc comparison of genotype × temperature treatments, and to assess genotypic differences in unreduced gamete production. Linear models were used to assess the relative significance of maternal parent, paternal parent, genotype and hybrid combination on unreduced gamete production.

## Competing interests

The authors declare that they have no competing interests.

## Authors' contributions

All authors contributed to conceptualization and experimental design. ASM carried out the experimental work and data analysis, and drafted the manuscript. WAC, MNN and GY supervised ASM and revised the manuscript. All authors read and approved the final manuscript.

## Supplementary Material

Additional file 1 **Cartoon of meiosis in a 2n = 2*x *= 2 dicotyledonous plant**. Cartoon of meiosis in a 2n = 2*x *= 2 dicotyledonous plant, showing sporad production observed at a) the end of normal meiosis, resulting in formation of a tetrad (4 reduced nuclei, n = *x *= 1) and b) meiosis with parallel spindles, resulting in the formation of a dyad (2 unreduced nuclei, n = 2*x *= 2). Click here for file

Additional file 2 **Detailed crossing record (buds pollinated, pod set and seed production) of interspecific hybridization success between one genotype of *B. juncea*, five genotypes of *B. napus *and two genotypes of *B. carinata***. Detailed crossing record (buds pollinated, pod set and seed production) of interspecific hybridization success between one genotype of *B. juncea*, five genotypes of *B. napus *and two genotypes of *B. carinata*. Click here for file

Additional file 3 **"Giant" pollen observations and unreduced and abnormal male gamete production in anomalous interspecific hybrids created between *Brassica napus, B. juncea *and *B. carinata***. "Giant" pollen observations and unreduced and abnormal male gamete production in anomalous interspecific hybrids created between *Brassica napus *(*B. n*: N1 and N3), *B. juncea *(*B. j*: J1) and *B. carinata *(*B.c*: C1). Four plants resulted from an unreduced female gamete from *B. napus *and a normal, reduced gamete from *B. juncea*. Another plant resulted from a normal, reduced female gamete of *B. juncea *and an abnormal (aneuploid, < n) gamete of *B. carinata*. Hypothetical "giant" pollen size in the hybrids was estimated from measurements of n and 2n pollen in *B. napus *and *B. juncea *under the assumptions that a) doubling DNA content would double pollen grain volume, and b) that reduced pollen in the hybrids would have a maximum DNA content of 4*x*. Both dyads and giant sporads were assumed to produce unreduced male gametes, whereas non-tetrad sporads were assumed to produce abnormal male gametes. Click here for file

Additional file 4 **Days to first flower in two *B. carinata *accessions (C1 and C2), one *B. juncea *accession (J1), two *B. napus *cultivars (N1 and N2) and in the interspecific hybrids between them (e.g. J1N1 = *B. juncea *J1 × *B. napus *N1) under four different temperature treatments**. Days to first flower in two *B. carinata *accessions (C1 and C2), one *B. juncea *accession (J1), two *B. napus *cultivars (N1 and N2) and in the interspecific hybrids between them (e.g. J1N1 = *B. juncea *J1 × *B. napus *N1) under four different temperature treatments. Temperature and genotype combined accounted for 95% of the variance in flowering time (p < 0.0001, r^2 ^= 0.95), with a small but statistically significant genotype × environment interaction (p < 0.05). Cold temperature significantly delayed flowering in 9/10 genotypes (p < 0.0001). Click here for file
