# Supplementary material for: Production of viable male unreduced gametes in Brassica interspecific hybrids is genotype specific and stimulated by cold temperatures
Source: BMC Plant Biol. 2011 Jun 12;11:103. doi: 10.1186/1471-2229-11-103 (PMC3141635; doi:10.1186/1471-2229-11-103)
Supplement: Additional file 1 — Cartoon of meiosis in a 2n = 2x = 2 dicotyledonous plant. Cartoon of meiosis in a 2n = 2x = 2 dicotyledonous plant, showing sporad production observed at a) the end of normal meiosis, resulting in formation of a tetrad (4 reduced nuclei, n = x = 1) and b) meiosis with parallel spindles, resulting in the formation of a dyad (2 unreduced nuclei, n = 2x = 2). [file 1471-2229-11-103-S1.PPT]

## Slide 1
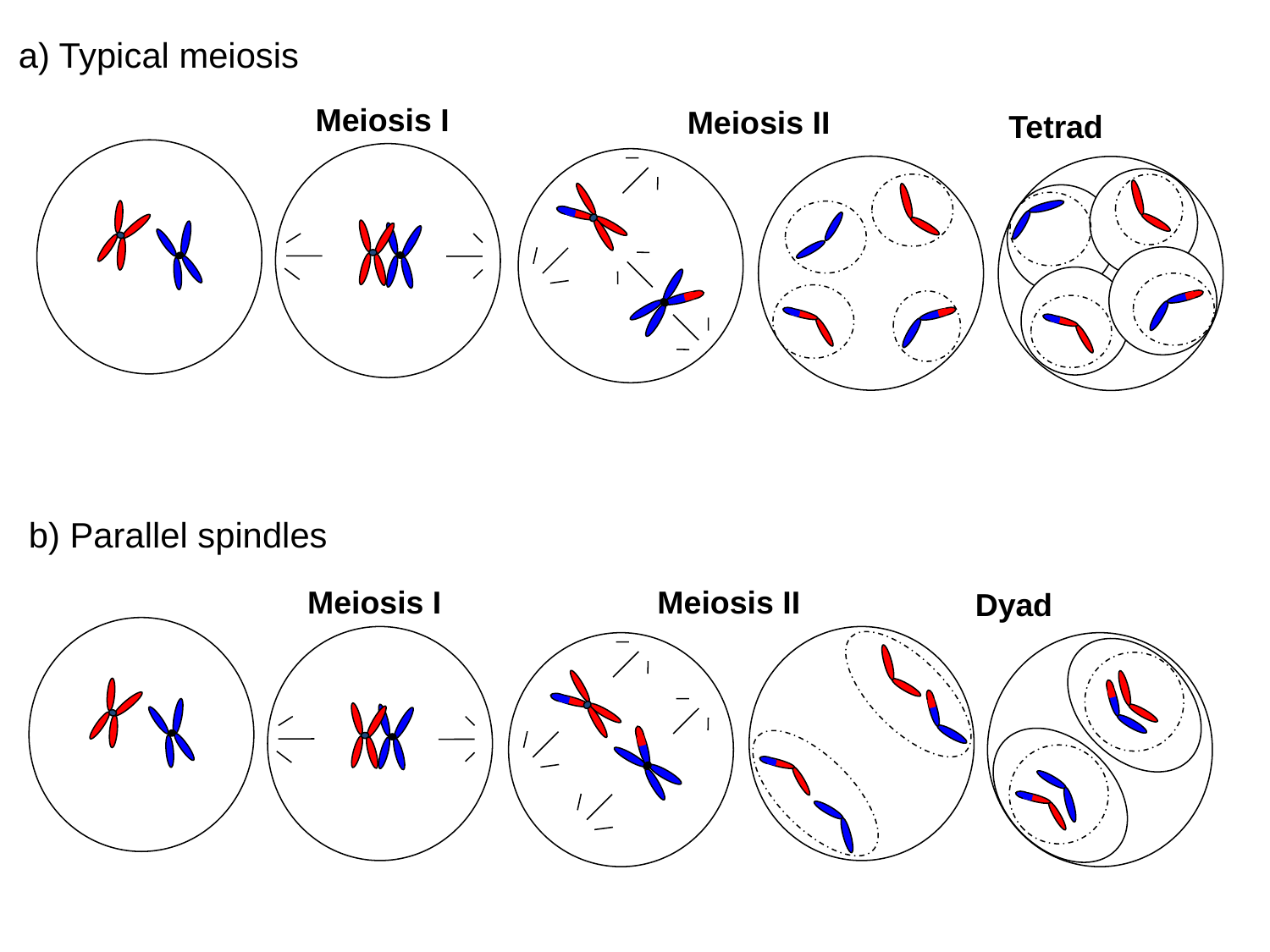

a) Typical meiosis
Meiosis I
Meiosis II
Tetrad
b) Parallel spindles
Meiosis I
Meiosis II
 Dyad
