# Supplementary material for: Production of viable male unreduced gametes in Brassica interspecific hybrids is genotype specific and stimulated by cold temperatures
Source: BMC Plant Biol. 2011 Jun 12;11:103. doi: 10.1186/1471-2229-11-103 (PMC3141635; doi:10.1186/1471-2229-11-103)
Supplement: Additional file 3 — "Giant" pollen observations and unreduced and abnormal male gamete production in anomalous interspecific hybrids created between Brassica napus, B. juncea and B. carinata. "Giant" pollen observations and unreduced and abnormal male gamete production in anomalous interspecific hybrids created between Brassica napus (B. n: N1 and N3), B. juncea (B. j: J1) and B. carinata (B.c: C1). Four plants resulted from an unreduced female gamete from B. napus and a normal, reduced gamete from B. juncea. Another plant resulted from a normal, reduced female gamete of B. juncea and an abnormal (aneuploid, < n) gamete of B. carinata. Hypothetical "giant" pollen size in the hybrids was estimated from measurements of n and 2n pollen in B. napus and B. juncea under the assumptions that a) doubling DNA content would double pollen grain volume, and b) that reduced pollen in the hybrids would have a maximum DNA content of 4x. Both dyads and giant sporads were assumed to produce unreduced male gametes, whereas non-tetrad sporads were assumed to produce abnormal male gametes. [file 1471-2229-11-103-S3.DOC]

| **Additional file 3: "Giant" pollen observations and unreduced and abnormal male gamete production in anomalous interspecific hybrids created between *Brassica napus* (*B. n:* N1 and N3), *B. juncea* (*B. j*: J1) and *B. carinata (B.c*: C1)*.*** | | | | | | | | | | | | |
| --- | --- | --- | --- | --- | --- | --- | --- | --- | --- | --- | --- | --- |
| Species cross | Hybrid genotype -genomes | No. plants | Average pollen viability | Total viable pollen measured | Giant pollen | **Giant pollen (% of viable pollen)** | Total sporads | Abnormal sporads | Abnormal male gametes (%) | Dyads | Giant sporads | **2n male gametes (%)** |
| *B. n* *x B. j* | J1N2 - AACCAB | 1 | 47% | 220 | 8 | **3.6%** | 327 | 27 | 9.6% | 0 | 0 | **0.00%** |
| *B. n* *x B. j* | J1N5 - AACCAB | 3 | 36% | 112 | 2 | **1.8%** | 607 | 7 | 1.2% | 1 | 0 | **0.08%** |
| *B. j x B. c* | J1C1 - AB + partial BC | 1 | 0% | 0 | - | **-** | 621 | 130 | 14.1% | 47 | 7 | **5.33%** |
|  | **Total** | **5** | **-** | **332** | **10** | **Av: 2.7%** |  |  |  |  |  |  |

Four plants resulted from an unreduced female gamete from *B. napus* and a normal, reduced gamete from *B. juncea*. Another plant resulted from a normal, reduced female gamete of *B. juncea* and an abnormal (aneuploid, <n) gamete of *B. carinata*. Hypothetical “giant” pollen size in the hybrids was estimated from measurements of n and 2n pollen in *B. napus* and *B. juncea* under the assumptions that a) doubling DNA content would double pollen grain volume, and b) that reduced pollen in the hybrids would have a maximum DNA content of 4*x*. Both dyads and giant sporads were assumed to produce unreduced male gametes, whereas non-tetrad sporads were assumed to produce abnormal male gametes.
