# Supplementary figures and images for: Production of viable male unreduced gametes in Brassica interspecific hybrids is genotype specific and stimulated by cold temperatures
Source: BMC Plant Biol. 2011 Jun 12;11:103. doi: 10.1186/1471-2229-11-103 (PMC3141635; doi:10.1186/1471-2229-11-103)

## Slide 1
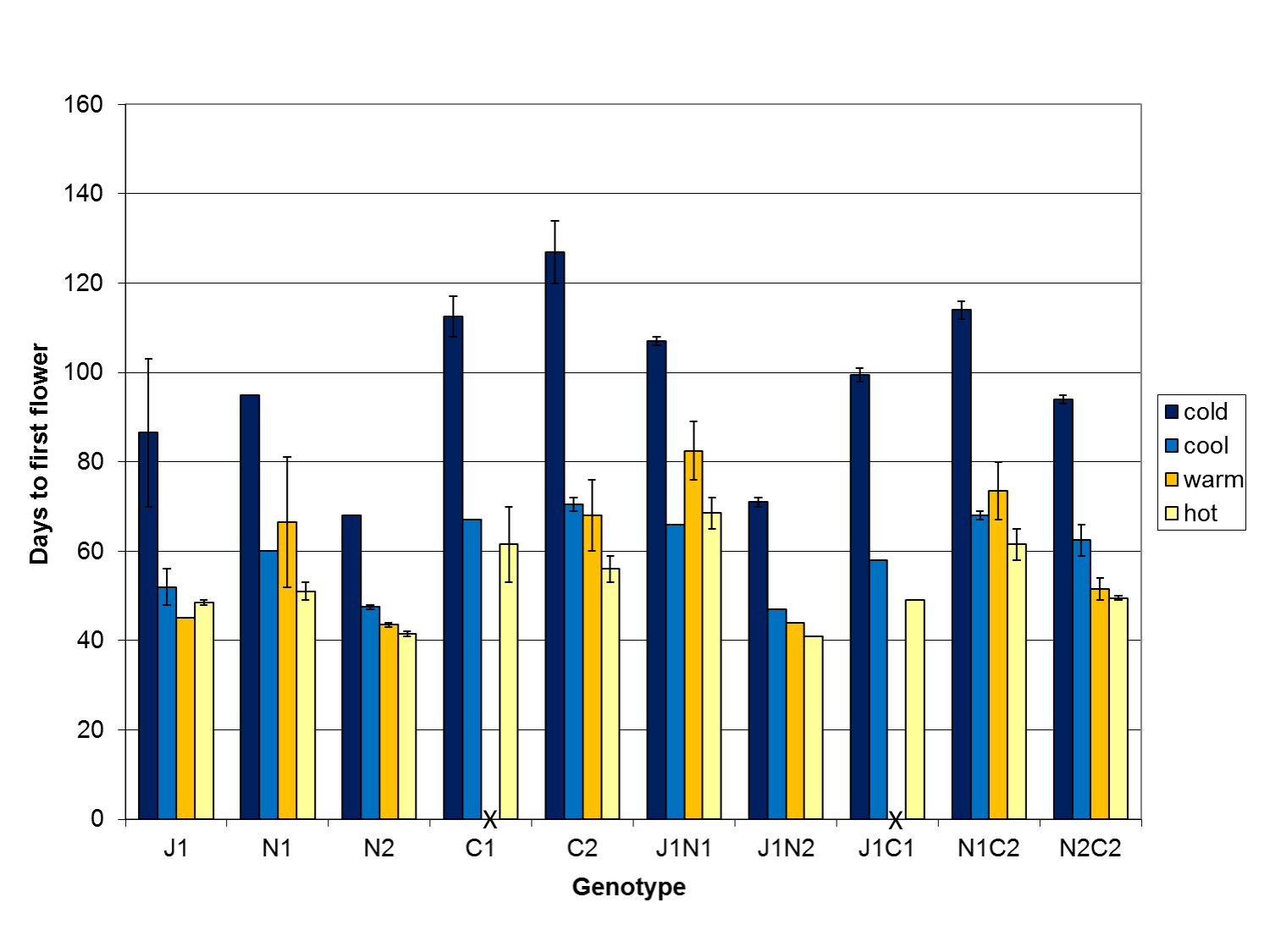

X
X

Supplement: Additional file 4 — Days to first flower in two B. carinata accessions (C1 and C2), one B. juncea accession (J1), two B. napus cultivars (N1 and N2) and in the interspecific hybrids between them (e.g. J1N1 = B. juncea J1 × B. napus N1) under four different temperature treatments. Days to first flower in two B. carinata accessions (C1 and C2), one B. juncea accession (J1), two B. napus cultivars (N1 and N2) and in the interspecific hybrids between them (e.g. J1N1 = B. juncea J1 × B. napus N1) under four different temperature treatments. Temperature and genotype combined accounted for 95% of the variance in flowering time (p < 0.0001, r2 = 0.95), with a small but statistically significant genotype × environment interaction (p < 0.05). Cold temperature significantly delayed flowering in 9/10 genotypes (p < 0.0001). [file 1471-2229-11-103-S4.PPT]
